# Implementation of a randomized mobile-technology lifestyle program in individuals with nonalcoholic fatty liver disease

**DOI:** 10.1038/s41598-024-57722-7

**Published:** 2024-03-28

**Authors:** Monica A. Tincopa, Nik Patel, Areesha Shahab, Haila Asefa, Anna S. Lok

**Affiliations:** 1 Division of Gastroenterology and Hepatology, Department of Internal Medicine, University of California San Diego, 9350 Campus Point Dr Ste 2B #0975, San Diego, CA USA; 2 Division of Gastroenterology and Hepatology, Department of Internal Medicine, University of Michigan, Ann Arbor, MI USA

**Keywords:** Exercise, Diet, NASH, NAFLD, eHealth, Liver diseases, Quality of life, Therapeutics, Weight management

## Abstract

Identifying effective, feasible, low-cost interventions that promote sustainable lifestyle changes in nonalcoholic fatty liver disease (NAFLD) is a key unmet need. The aim of this study was to assess predictors of lifestyle practice patterns of NAFLD patients and evaluate the implementation of a mobile technology-based intervention. We prospectively enrolled adults with NAFLD (diagnosed by imaging or biopsy). Individuals with additional liver diseases or decompensated cirrhosis were excluded. Patient were randomized to usual care or a FitBit based program for 6-months. We obtained anthropometrics, labs, vibration controlled transient elastography (VCTE), health-related quality of life (HRQOL), physical activity, diet and motivation to change data. 70 patients were enrolled, 33% with cirrhosis. Median age was 52.1 years, 47% males, 83% white, body mass index 32.3, liver stiffness 7.6 kPa, controlled attenuation parameter 319 db/m, and 50% had diabetes. Baseline HRQOL was 5.4/7 and independently negatively correlated with level of concern about their disease and positively with physical function. Younger age was independently associated with unhealthy diets whereas diabetes was independently associated with unhealthy diets and higher VCTE kPa. 6-month follow-up data available on 31 patients showed trends in improvement in weight. In a cohort of NAFLD patients, we identified independent correlates of lifestyle behaviors and HRQOL. Implementation of interventions that improve physical function may improve HRQOL in NAFLD. Younger patients and those with diabetes appeared to have the greatest need for dietary interventions. Structured mobile technology lifestyle interventions using Fitbit and personalized coaching showed promise but require further validation with a focus on sustainability of intervention and improvement in outcomes.

## Introduction

An estimated 1 billion individuals have underlying nonalcoholic fatty liver disease (NAFLD)^1^. Its more aggressive subtype, nonalcoholic steatohepatitis (NASH) is associated with risk of progression to cirrhosis, liver cancer and need for liver transplantation^2^. Given increasing global prevalence of NAFLD, identifying and implementing effective and scalable therapy is critical to optimize clinical and patient-reported outcomes. There is exciting progress in developing effective pharmacotherapy for NASH, though none have been approved by the Food and Drug Administration (FDA) so far. In parallel there have been notable advances in pharmacotherapy for the treatment of obesity and several agents have been approved for clinical use^3^. These agents are relevant to clinical care of NAFLD given that weight loss has been shown to be associated with improvement in liver histology and clinical outcomes; however, these medications are expensive and their long-term safety and efficacy are uncertain^4^.

A key challenge in clinical practice remains identification and implementation of effective, low cost and sustainable programs to promote healthy eating and regular exercise to achieve weight loss in patients with NAFLD. There has been interest in mobile technology-based interventions given that this design can avoid the need for travel, decrease time commitment, and provide an option for real-time feedback^5^. A recent systematic review and meta-analysis found that eHealth technologies are effective for improvements in body mass index (BMI) and liver enzymes in patients with NAFLD^6^. While these types of programs have shown promise, very few studies have been done in the United States (US) and these cohorts included no more than 40 participants. We previously showed the feasibility and acceptability of a mobile technology based lifestyle intervention in patients with NAFLD and found that the program promoted physical activity and improvements in clinical, metabolic and hepatic parameters in a subset of participants^7^. In this study, we address an important knowledge gap regarding implementation of these programs among larger cohorts in general clinical practice in order to inform evidence-based practices in real-world settings and provide data on effectiveness of these interventions. Herein we implemented an enhanced version of our previous mobile technology based lifestyle program and compared it to usual care in our hepatology clinic. The aims of this study were to assess a comprehensive list of diverse factors associated with lifestyle practice patterns of patients with NAFLD and to evaluate the implementation of a mobile technology-based intervention.

## Methods

### Patient population

The design of this study was modeled off of our initial pilot intervention previously described^7^. We prospectively enrolled 70 adult patients with a diagnosis of NAFLD from our hepatology outpatient clinic in Ann Arbor, Michigan between April 2019 and March 2020 with follow-up through August 2020. To meet diagnostic criteria for NAFLD, a participant was required to have imaging [ultrasound (US), Vibration controlled transient elastography (VCTE) (Fibroscan, Echosens), computed tomography (CT), or Magnetic Resonance Imaging (MRI)] demonstrating steatosis within the prior 24 months or a liver biopsy noting hepatic steatosis within the prior 36 months, with no or minimal weight loss (< 5%) since those tests. This testing window was selected to minimize the need for repeating previously performed tests to qualify for entry into the study. Ten subjects had imaging or biopsy completed 6-months or more prior to enrollment with the remaining having testing completed within 6-months prior to enrollment. Patients with any other additional cause of chronic liver disease such as hepatitis B or C were excluded. Alcohol assessment was conducted based on chart review and participant response to query during screening visit. Patients who reported > 14 drinks per week for males or > 7 drinks per week for females at time of their screening visit or with any prior history of alcohol use disorder were excluded. All participants were required to be able to participate in a walking program and basic nutritional interventions (able to follow a Mediterranean or low carbohydrate diet). Those with severe medical co-morbidities (i.e., severe cardiopulmonary disease, severe musculoskeletal disease, uncontrolled diabetes, active malignancy), hepatic decompensation, prior liver transplant, or hepatocellular carcinoma were excluded. Participants with compensated cirrhosis were eligible for enrollment given the overall systemic health benefits of healthier lifestyle habits in addition to data supporting the role of lifestyle interventions in this population in reducing risk of clinical decompensation including reductions in degree of portal hypertension^8^. Individuals receiving medications that may cause hepatic steatosis or weight reduction, and those who had plans for bariatric procedures or enrollment in other structured lifestyle programs were also excluded. Participants were required to have access to a computer or a smartphone with internet connection.

### Data collection

At enrollment we obtained data on demographics, medical comorbidities, vital signs and anthropometrics, laboratory studies (up to 6 months from time of enrollment), physical function and frailty measures, hepatic imaging and several survey measures. Physical function was assessed using the 6-minute walk test (6MWT). The 6MWT is an efficient, low-cost method to assess functional exercise capacity that has been validated in individuals with chronic liver disease^9^. Frailty was assessed using hand grip strength via dynamometry according to established protocols by trained research staff. Three measurements were made with each hand and the dominant hand was noted. VCTE liver stiffness (LSM) and controlled attenuation parameter (CAP) measurements were obtained at baseline unless results of VCTE performed within 6 months prior to enrollment were available and participant did not have ≥ 5% weight loss since that exam.

Physical activity was assessed using the validated short-version International Physical Activity Questionnaire (IPAQ)^10^. Dietary assessment was conducted using the Starting the Conversation survey which has been used extensively as a concise measure of healthy eating^11^. This is an eight question tool that assessed the frequency of intake of fast food, vegetables, fruits, sugar-sweetened beverages, low-fat and lean proteins, chips and crackers, desserts/other sweets and margarine/butter/meat fat. Each response is scored from 0 to 2 for a maximum of 16 points, with higher scores indicating unhealthy dietary habits. Health-related quality of life (HRQOL) measures were obtained using the Chronic Liver Disease Questionnaire-NAFLD (CLDQ-NAFLD)^12^. This instrument consists of 36 items over six domains: fatigue, abdominal symptoms, emotional function, systemic symptoms, activity, and worry. Each question requires a response on a Likert-scale from 1–7 to indicate “all of the time” to “none of the time”. Responses to these items are averaged to give a summary score between 1 and 7 with higher scores indicating higher HRQOL. Motivation to change health behaviors was evaluated using the validated Stages of Change questionnaire^13^. This model categorizes readiness to change health behaviors into one of 5 categories: (1) pre-contemplation; (2) contemplation; (3) preparation; (4) action; and (5) maintenance.

At month 6, repeat anthropometrics, laboratory studies, and surveys were conducted. Follow-up measures were limited by the impact of the COVID-19 pandemic that restricted in person research visits and data collection. Participants received a $25 gift card for each study visit completed. Procedures of the study were approved by the University of Michigan Institutional Review Board and all participants provided informed written consent prior to the study. All methods were performed in accordance with relevant guidelines and regulations. This study was first registered in clinicaltrials.gov on 12/02/2019 (NCT03839082). Results of the study are reported in accordance with the CONSORT 2010 guidelines.

### Lifestyle intervention

**Figure 1:**
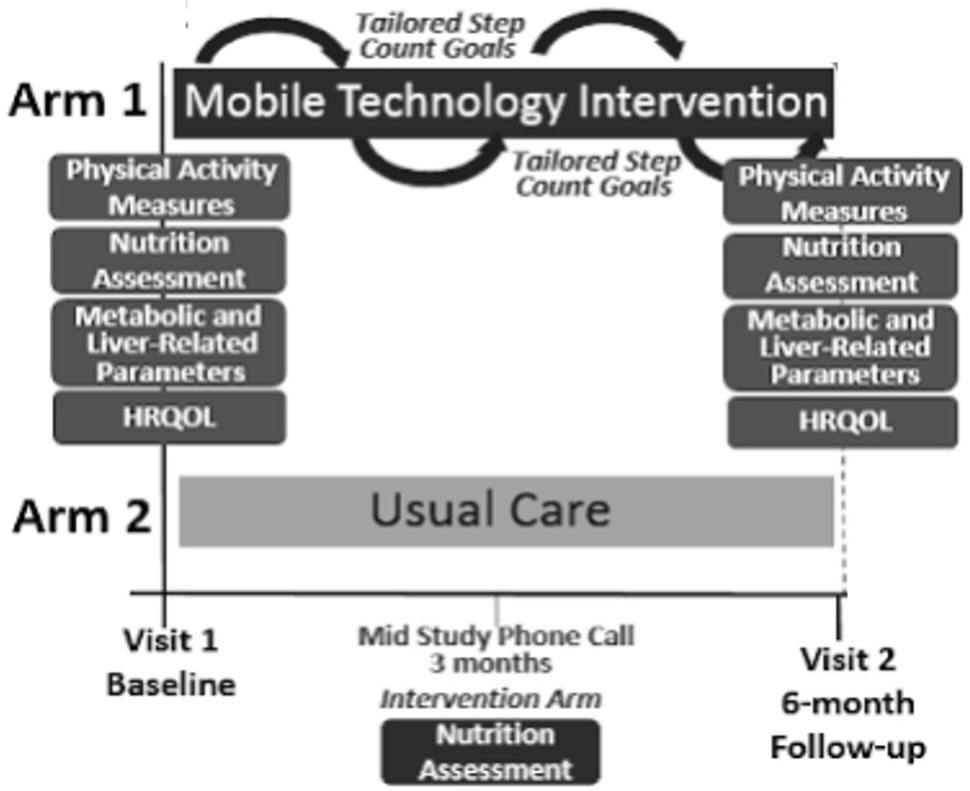
Mobile-technology based intervention compared to usual care.

Patients were randomized to either usual care in the general hepatology clinic or to mobile-technology based intervention for 6-months (Fig. 1). At our center, usual care for patients with NAFLD without decompensated cirrhosis consists of visits every 6–12 months. Visits are approximately 15–20 min and consist of review of weight, recent laboratory tests and VCTE/imaging if ordered. Management typically consists of a brief overview of lifestyle changes including improvements in nutrition and physical activity. Patients in usual care did not consistently receive specific educational materials nor dietician evaluation. Vitamin E is prescribed in a small proportion of patients. A similarly small proportion of patients are formally referred to and subsequently evaluated by a nutritionist.

The mobile technology-based lifestyle intervention design was informed by our prior pilot trial^7^. Participants in the intervention arm received a Fitbit Zip at enrollment to track step counts. The Fitbit wirelessly syncs data from the tracker to the Fitbit software or app. Study staff assisted with downloading the software and instructed participants to wear their FitBit during waking hours every day. At any time if the participant had questions or problems regarding the use of the FitBit, a study staff member could be contacted.

Study staff retrieved users’ step count data for analysis weekly and provides subjects with personalized feedback on step counts with tailored goals (10% increase per week with maximum increase of 800 steps per week to a maximum of 10,000 steps/day) and motivational messaging based on their prior step count and nutritional evaluation via e-mail. These goals were modeled after the United States Preventative Services Task Force (USPSTF) recommendations for physical activity^14^. Patients with consecutive days without data recorded or with other signs of low FitBit usage (days with minimal step counts) were contacted by study staff via e-mail or phone if necessary to encourage use. Feedback occurred weekly for the first 3 months and then biweekly for another 3 months. Patients in the intervention arm also had a nutritional assessment by a nutritionist specialized in NAFLD at enrollment. As part of the longitudinal feedback e-mails, patients were also asked about progress with diet/nutrition. Participants in the intervention arm also received our NAFLD educational folder that included: (1) NAFLD disease information including diagnosis, clinical manifestations, natural history and treatments; (2) NAFLD nutritional recommendations including sample menus; (3) NAFLD physical activity recommendations including walking programs and physical activity logs; (4) weight tracking logs; and (5) resources for diet and exercise programs^15^. Participants were encouraged to incorporate physical activity beyond walking. Data on types of physical activity completed by the participant on a regular basis were captured in the IPAQ assessments.

### Outcomes of interest

The outcomes included correlates of lifestyle behaviors and HRQOL, and improvements in metabolic and liver-related clinical parameters, HRQOL, and physical activity patterns at month 6 after implementation of our mobile technology intervention. Due to COVID-19 pandemic restrictions on in-person clinic and research visits, follow-up data was restricted to survey data which limited the pre/post analyses of the impact of the intervention.

### Statistical analysis

To assess baseline characteristics and impact of the intervention on outcomes of interest we performed descriptive and bivariate analyses. For descriptive statistics, medians and interquartile ranges (IQR) for continuous data and frequencies and percent for categorical data are presented. Correlations between lifestyle patterns and variables of interest were determined by univariate and multivariate linear and logistic regression. Candidate variables for multivariate analyses were selected based on results of univariate analysis, biologic plausibility and results of prior published studies. End of intervention analyses were assessed using the Wilcoxon rank-sum test to evaluate differences in medians given the small sample size and wide distribution of data points in the cohort. *P* values < 0.05 were considered statistically significant. All analyses were performed in STATA 14 (StataCorp, College Station, TX).

## Results

### Patient population

A total of 70 patients were enrolled. Baseline characteristics of participants are shown in Table 1. The median age of the cohort was 52.1 years with 47.1% were males and 83% white. Overall, 35% had diabetes, 46% had hyperlipidemia and median BMI was 32.2. Twenty-two patients had compensated cirrhosis. At enrollment, median liver stiffness was 7.6 kPa with 45.9% categorized as F ≥ 3, and median CAP was 319 db/m. Overall, 29 (41.4%) participants met the USPSTF recommendations for an average of 150 min/week of moderate or 75 min/week of vigorous physical activity. For baseline physical function, the median distance walked in the 6MWT was 1,475 feet (IQR 1315–1690) with the general population average for healthy middle age adults being 1620–1890 feet.^16,17^ Median frailty measures based on dominant hand grip strength was 76.2 lb for males and 55.1 lb for females with the average dominant grip strengths in the general population for this cohort’s median age range being 97 lb for men and 62.2 lb for women.^18,19^ Median dietary score was 7 (range 0–16, with higher scores indicating less healthy diets). Baseline HRQOL was 5.4 (range 0–7, with higher scores indicating better HRQOL). Overall, 45% and 68% of participants reported being in the action/maintenance phase for change toward healthy physical activity and diet, respectively with the majority of participants acknowledging high importance of behavior change.

**Table 1:** Baseline characteristics.

Characteristics	N = 70	
Age	52.1 (43–63)	
Race, white	57 (82.6%)	
Sex, male	33 (47.1%)	
BMI	32.2 (29.2–37.1)	
Waist circumference (in)	44 (40–47.5)	
Diabetes	35 (50%)	
Hyperlipidemia	32 (46.4%)	
Cirrhosis	22 (32.8%)	
ALT (U/L)	47 (30–70)	
Triglyceride (mg/dL)	137 (110–232)	
Hemoglobin A1c	6 (5.6–7.2)	
VCTE liver stiffness kPa^@^	7.6 (5.7–14.8)	
> 9 kPa	22 (45.9%)	
VCTE CAP (db/m)^@^	319 (285–353)	
CLDQ-NAFLD*	5.5 (4.6–6.1)	
Vigorous Activity, Days/wk; min/day	1 (0–3); 17.5 (0–30)	
Moderate Activity, Days/wk; min/day	1 (0–3); 30 (17.5–35)	
6MWT (feet)	1475 (1315–1690)	
Grip strength (lb)		
Males	76.2 (47.3–98)	
Females	55.1 (43.1–63.1)	
Diet Score (max 16)^	7 (5–9)	

	Physical activity	Diet
Stages of change model		
Not intending to change in the next 6 months	2 (3%)	4 (5.9%)
Intend to change in the next 6 months	22 (32.8%)	10 (14.9%)
Intend to change in the next 1 month	13 (19.4%)	14 (20.9%)
Already changed behavior	18 (26.9%)	22 (32.8%)
Working to maintain new behavior	12 (17.9%)	17 (35.4%)
Importance of Behavior Change (max 10)#	9 (8–10)	9 (7–10)
Level of Concern about NAFLD#	7 (5–9)	

Data presented as median (interquartile range) or number (percent)

VCTE, vibration controlled transient elastography; CAP, controlled attenuation parameter; CLDQ, Chronic Liver Disease Questionnaire; NAFLD, Nonalcoholic Fatty Liver Disease.

6MWT, 6 min walk test; ^@^VCTE data available in 49 patients only; *Higher scores indicate higher HRQOL (max score 7); ^Higher scores indicate unhealthy diets (max score 16);

^#^0 being not important/no concern and 10 being very important/concerned.

### Predictors of lifestyle behaviors and HRQOL

We assessed correlates of HRQOL, lifestyle behaviors, motivation to change and stages of hepatic disease to identify variables impacting disease management and outcomes (Table 2). On univariate analysis there were several variables associated with HRQOL, though only 6MWT distance (coefficient 0.001, p 0.01) and concern about disease (coefficient −0.09, *p* 0.02) remained statistically significant on multivariate analysis. There were no correlates of physical activity (defined as total minutes spent in vigorous plus moderate physical activity by self-report). Higher self-reported diet scores (indicating unhealthy diets) were independently negatively correlated with age (coefficient −0.06, *p* 0.01) and positively correlated with diabetes (coefficient 1.75, *p* 0.02). The only variable independently correlated with active stage of change for physical activity was a concomitant active stage of change for diet (coefficient 1.75, *p* 0.004). Individuals in an active stage of change for diet had lower baseline diet scores (indicating healthier diets) (coefficient −0.25, *p* 0.01).

**Table 2:** Multivariate associations with baseline HRQOL and lifestyle patterns.

Variable	Univariate		Multivariate	
	Coeff (95% CI)	*P* value	Coeff (95% CI)	*P* value
CLDQ-NAFLD				
Age	−0.02 (−0.03, −0.00)	0.03	−0.00 (−0.02–0.12)	0.59
Sex (male)	0.57 (0.05–1.08)	0.02	0.32 (−0.12–0.77)	0.15
BMI	−0.03 (−0.07, 0.12)	0.15		
Waist Circumference	−0.35 (−0.07–0.01)	0.11		
Cirrhosis	−1.04 (−1.58, −0.49)	< 0.001	−0.47 (−1.04–0.09)	0.10
Diabetes	−0.15 (−0.68, 0.37)	0.56		
VCTE CAP	−0.00 (−0.01, 0.00)	0.76		
Total vigorous and moderate physical activity	0.00 (−0.00–0.00)	0.34		
Hand grip	0.01 (−0.00–0.15)	0.18		
6MWT	0.00 (0.00–0.00)	< 0.001	0.001 (0.00–0.00)	**0.01**
Diet score	−0.00 (−0.09–0.09)	0.95		
Concern about disease	−0.13(−0.21, −0.04)	0.002	−0.09 (−0.17, −0.15)	**0.02**
Physical activity (total minutes of moderate and vigorous combined)				
Age	3.81 (−0.19–7.83)	0.06	2.63 (−1.55–6.82)	0.21
Sex (male)	2.38 (−115.58–120.35)	0.96		
BMI	−7.31 (−15.70–1.06)	0.08	−4.51 (−13.79–4.76)	0.33
Waist Circumference	−4.91 (−14.11–4.27)	0.28		
VCTE CAP	0.23 (−1.16–1.62)	0.73		
VCTE liver stiffness	−2.56 (−10.68–5.55)	0.52		
Hand grip	0.47 (−1.74–2.70)	0.66		
Cirrhosis	40.58 (−81.28–162.45)	0.50		
Diabetes	22.68 (−97.42–142.79)	0.70		
Diet score	−9.81 (−31.28–11.6)	0.36	2.73 (−19.26–24.73)	0.80
Concern about disease	−4.64 (−23.99–14.70)	0.63		
Diet score				
Age	−0.05 (−0.10, −0.01)	0.02	−0.06 (−0.11, −0.01)	**0.01**
Sex (male)	−0.38 (−1.77–1.06)	0.58		
BMI	0.10 (−0.01–0.20)	0.06		
Waist Circumference	0.11 (−0.00–0.22)	0.05	0.01 (−0.10–0.14)	0.74
VCTE CAP	0.01 (−0.01–0.02)	0.30		
VCTE kPa	0.06 (−0.03–0.16)	0.19		
Cirrhosis	1.04 (−0.40–2.49)	0.15		
Diabetes	1.59 (0.23–2.95)	0.02	1.75 (0.32–3.17)	**0.02**
6MWT	−0.00 (−0.00–0.00)	0.55		
Hand grip	−0.00 (−0.02–0.02)	0.75		
Concern about disease	−0.07 (0.30–0.15)	0.51		
Active stage of change: physical activity				
Age	0.02 (−0.01–0.06)	0.15	0.02 (−0.12–0.07)	0.16
Sex (male)	−0.21 (−2.28–0.75)	0.66	−0.40 (−1.54–0.73)	0.48
BMI	−0.17 (−0.09–0.06)	0.66		
Cirrhosis	−0.41 (−1.47–0.63)	0.44		
Diabetes	0.15 (−0.82–1.12)	0.31		
Hand grip	−0.00 (−0.01–0.15)	0.81		
6MWT	0.00 (−0.00–0.00)	0.12	0.00 (−0.00–0.00)	0.11
CLDQ-NAFLD	0.39 (−0.13–0.91)	0.14		
Diet score	−0.01 (−0.19–0.15)	0.85		
Active Stage of Change: Diet	1.76 (0.65–2.87)	0.002	1.75 (0.55–2.95)	**0.004**
Concern about disease	0.05 (−0.10–0.21)	0.48		
Active stage of change: diet				
Age	0.02 (−0.01–0.05)	0.20	0.01 (−0.02–0.04)	0.64
Sex (male)	−0.25 (−1.23–0.71)	0.60		
BMI	0.01 (−0.06–0.08)	0.81		
Cirrhosis	−0.42 (−1.4–0.61)	0.42		
Diabetes	−0.28 (−1.2–0.69)	0.56		
CLDQ-NAFLD	−0.03 (−0.54–0.46)	0.87		
Diet score	−0.25 (−0.45, −0.05)	0.01	−0.25 (−0.47, −0.04)	**0.01**
Concern about disease	0.08 (−0.07–0.24)	0.31	1.42 (−1.51–4.35)	0.34

Significant values are in bold.

### Intervention uptake and retention

Given that enrollment and follow-up of this study occurred during the peak of the COVID-19 pandemic which significantly impacted health care practice patterns and general population psychology and health behaviors, participants were unable to return for in-person research visits. In addition, a high proportion of participants did not complete follow-up data assessments that could be collected remotely. Participants were not required to provide reasons for study discontinuation, but responses included competing health issues, time constraints, and decisions to not continue in research during the pandemic. As a result, we had follow-up data available on 31 patients consisting of survey data and self-reported follow-up weights (Table 3). There was insufficient reliable step-count data to statistically analyze pre-post change in step count. Among those with available valid step count data, at baseline the median step count per day was 3067 (IQR 1153–6453). At month 3 the median daily step count was 3980 (IQR 1677–5705) and at month 6 was 4840 daily (IQR 3325.25–6720.75), though few participants had reliable step count data at the end of the intervention. There were trends in improvement in weight, VCTE kPa and CAP scores at study completion, though not at a statistically significant level. There were no statistically significant differences at 6-months between the intervention arm and usual care, though these analyses were limited due to small sample size.

**Table 3.** 6-Month follow-up data.

Variable	Baseline	6-Month	Median (IQR) Pre-post	*P* value
Overall cohort (N = 31)				
Weight, lb (N = 31)	205 (181.6–266.3)	195 (183–268)	−0.5 (−0.74, 0)	0.15
VCTE (N = 5) LSM (kPa)	7.8 (6.2–8.3)	6.7 (5.4–11)	−1 (−2.9, 1)	0.58
VCTE CAP (db/m) (N = 5)	344 (338–388)	325 (305–365)	−35 (−63, 10)	0.34
CLDQ-NAFLD* (N = 15)	5.47 (4.3–6.5)	5.52 (4.75–6.36)	0.16 (−0.83, 0.55)	0.15
IPAQ (N = 17)				
Vigorous activity	1 (0–3);	0 (0–2);	0 (0–2);	0.09
Days/wk; min/day	20 (5–37.5)	20 (15–30)	17.5 (−5, 30)	0.17
Moderate activity	1 (0–3);	1 (0–2)	0 (−1, 1);	0.80
Days/wk; min/day	30 (30–45)	30 (30–30)	0 (0–237)	0.31
Walking	5 (3–7);	5 (2–7);	0 (−1, 1);	0.88
Days/wk; min/day	30 (20–30)	30 (15–30)	0 (−30, 10)	0.66
Sitting hr/day	6.5 (4.5–10)	7 (4–12)	0 (−1.5, 2)	0.81
Diet score (N = 12)	7 (6–8.5)	5.5 (3.5–7.5)	−2 (−3.5, 0.5)	0.07
Importance of behavior change (N = 6)				
Physical activity	9.5 (8–10)	10 (9–10)	0.5 (0, 1)	0.36
Diet	9.5 (8–10)	9.5 (9–10)	0.5 (0, 1)	0.31
Level of concern about NAFLD (N = 6)	9 (8–10)	10 (5–10)	0 (0–0)	0.89
Intervention Arm (N = 17)				
Weight, lb (N = 16)	217.9 (181.6–282.2)	191 (183–280)	0 (0, 0.2)	0.30
VCTE (N = 1) LSM (kPa)	7.8	11	3.2	n/a
VCTE CAP (db/m) (N = 1)	388	382	−6	n/a
CLDQ-NAFLD (N = 5)	4.9 (4.4–6.5)	5.3 (4.9–5.6)	0.25 (−0.1, 0.3)	0.17
IPAQ (N = 6)				
Vigorous activity	3 (1–5);	1 (0–4);	−1.5 (−2, 0);	0.16
Days/wk; min/day	60 (60–60)	7.5 (0–60)	0 (−60, 0)	0.16
Moderate Activity	0.5 (0–4);	2 (0–7);	1 (−1, 2);	0.78
Days/wk; min/day	15 (0–120)	30 (3–30)	0 (−90, 30)	0.78
Walking	4 (3–7);	6 (2–7);	0 (−1, 0);	1.0
Days/wk; min/day	40 (20–60)	30 (30–80)	17.5 (12.5, 54)	0.31
Sitting hr/day	7 (6–10)	6 (3–12)	−1 (−3, −1)	0.65
Diet Score (N = 0 for pre/post)	n/a	n/a	n/a	n/a
Importance of behavior change (N = 3)				
Physical activity	8 (5–9)	10 (9–10)	2 (2, 5)	0.10
Diet	8 (5–9)	9 (6–10)	1 (1, 1)	0.08
Level of concern about NAFLD (N = 3)	8 (5–10)	10 (5–10)	0 (0, 2)	0.31
Usual care arm (N = 14)				
Weight, lb (N = 15)	205 (161.5–225.3)	195.5 (183–222)	−0.6 (−1.2, 0.4)	0.40
VCTE (N = 4) LSM (kPa)	7.25 (5.95–19.8)	6.05 (5.3–14)	−1.95 (−6.5, 0)	0.17
VCTE CAP (db/m)	366 (319.5–394)	325 (286.5–345)	−49 (−69.5, 12.5)	0.65
CLDQ-NAFLD (N = 10)	5.6 (4.3–6.0)	5.6 (4.7–6.4)	0,01 (−0.1, 0.6)	0.18
IPAQ (N = 11)				
Vigorous Activity	0 (0–3)	0 (0–2)	0 (−1, 0)	0.16
Days/wk; min/day	0 (0–60)	0(0–60)	0 (0, 60)	1.0
Moderate Activity	60	30	−150	0.31
Days/wk; min/day	(0–180)	(10–30)	(−237, 10)	0.17
Walking	5 (3–7)	5 (1–7)	0 (−2, 2)	1.0
Days/wk; min/day	37 (30–60)	60 (25–120)	−15 (−32, 0)	0.31
Sitting hr/day	6 (4–10)	7 (6–12)	1 (−1,2)	0.59
Diet Score (N = 12)	7 (6–8.5)	5.5 (3.5–7.5)	−2 (−3.5, 0.5)	0.59
Importance of behavior change (N = 3)				
Physical activity	10 (10–10)	10 (8–10)	0 (−2, 0)	0.47
Diet	10 (10–10)	10 (9–10)	0 (−1, 0)	0.47
Level of concern about NAFLD (N = 3)	10 (8–10)	10 (4–10)	0 (0–4)	0.47

## Discussion

Achieving 5–10% weight loss has been shown to be associated with histological improvement in patients with NAFLD but this goal is rarely accomplished with counseling on nutrition and physical activity^20^. Even after FDA-approval pharmacotherapy, identification of scalable mechanisms to augment lifestyle changes will remain central to management of NASH. Obesity pharmacotherapy and bariatric procedures have been shown to decrease NASH activity and fibrosis but not all patients are eligible for these therapies and not all patients achieve or maintain weight loss with these therapies^3,21,22^. Even for patients who are eligible for these therapies, maintenance of healthy nutrition and regular physical activity are essential to prevent weight regain. Many studies have demonstrated the benefit of healthy eating and exercise on hepatic steatosis even in the absence of weight loss^23,24^. Emerging data also suggest that exercise alone even in the absence of significant weight loss may improve hepatic inflammation and liver stiffness measurements^25^. While smaller clinical trials have shown improvements in BMI and hepatic markers from mobile technology based lifestyle intervention programs in NAFLD, there is a paucity of data focused on implementation of mobile technology based lifestyle interventions in real-world clinical practice and thus effectiveness of such programs. In our study, we demonstrate the potential challenges and benefits of these programs in a larger cohort of patients with NAFLD cared for in a general hepatology clinic. Our study also provides useful data that builds on the existing literature by evaluating a comprehensive array of patient characteristics that may impact lifestyle change (including HRQOL, motivation to change, physical function/frailty) and application of validated dietary and physical activity assessments.

Patients with NAFLD including those without cirrhosis have impaired HRQOL^26^. In an earlier educational intervention study, we found that male sex, lower degree of formal educational training and higher BMI were associated with lower CLDQ-NAFLD scores^15^. In our current study, when additional measures of physical function and frailty were incorporated, only 6MWT distance and level of concern about disease emerged as independent correlates of baseline HRQOL. Our results are consistent with that of the NASH Clinical Research Network (CRN) that found a strong relationship between HRQOL and physical health, highlighting the importance of interventions to improve physical functioning in addition to improvements in metabolic and hepatic measures^26^. In this study we did not identify any factors independently associated with total self-reported moderate and vigorous physical activity, though in our prior educational study male sex was negatively correlated. A prior survey-based study of 87 patients with NAFLD by Stine et al. found that lack of exercise resources and education, time constraints and physical discomfort were associated with lower rates of exercise^27^. These findings were echoed in another survey-based study wherein lack of resources was a significant predictor of lower physical activity levels in individuals with NAFLD^27^.

**Figure 2:**
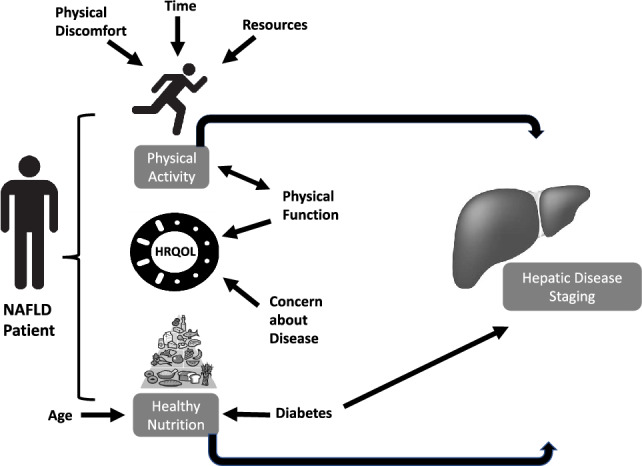
Conceptual framework of factors driving lifestyle behaviors and disease characteristics in NAFLD.

There is substantial literature assessing dietary habits and risk of NAFLD and NASH, but there is limited data evaluating predictors of healthy eating habits in these patients. We found that older individuals had healthier self-reported dietary habits (coefficient −0.06, *p* 0.01) and those with diabetes had less healthy dietary habits (coefficient 1.75, *p* 0.02). Our findings highlight the importance of understanding demographic, social and clinical variables that may make healthy eating patterns more challenging, as these factors may help identify those who require more intensive dietary interventions and tailor recommendations to overcome barriers each of these groups face. Figure 2 represents a conceptual framework building on our prior work and other published data on this area to demonstrate relevant factors driving lifestyle behaviors and disease characteristics in NAFLD^28^.

Our original intention was to determine the comparative effectiveness of usual care to a mobile technology-based lifestyle intervention on change in weight, liver, and metabolic parameters among patients with NAFLD. Unfortunately, this study was conducted during the peak of the COVID-19 pandemic which significantly impacted usual care and lifestyle patterns of patients. Among the 31 patients who had available 6-month follow-up weights, there was an overall trend in improvement in weight irrespective of study arm, though not at a statistically significant level. Similarly, among the small number of patients with follow-up HRQOL assessments, we saw trends in improvements, albeit small interval changes. Mobile technology-based lifestyle interventions have shown efficacy in improving lifestyle habits and weight loss in both the general obesity and diabetes populations, and emerging data has also shown promise in NAFLD cohorts^6^. Our study highlights challenges associated with implementation of these programs among patients with NAFLD, though future studies implemented beyond the COVID pandemic timeframe are necessary to identify which challenges were unique to the pandemic versus the patient population and/or study design features.

It is important to highlight several limitations of our study. Foremost, while our study cohort was substantially larger than prior RCTs of mobile technology lifestyle interventions in the US, the follow-up data was not powered to assess efficacy of the FitBit intervention. In addition, this was a single center study with a homogenous patient population consisting of predominantly white participants, limiting the generalizability of our findings. Given the demographics of our patient population, we did not have non-English speakers eligible for enrollment from our recruitment pool. Implementation in a more heterogeneous patient population including non-English speakers needs to be evaluated in future studies to address the fidelity of this approach as a mechanism to optimize first-line therapy for NAFLD. Furthermore, patients who were willing to enroll in a structured lifestyle intervention program may have different baseline disease characteristics and motivation to change lifestyle behaviors which potentially may also impact our cohort representativeness. Finally, the COVID pandemic hit during the peak follow-up period for our study which impacted retention and follow-up data collection. In particular, we cannot speak to sustainability of the intervention given our available data in the setting of attrition. Given the design of our intervention, it is also difficult to isolate the impact of various features of the program including the FitBit vs other lifestyle counseling and follow-up. A recent systematic review does suggest effectiveness of electronic-based lifestyle interventions in NAFLD on multiple metabolic parameters including weight, BMI, waist circumference and liver enzymes, though durability of these improvements remains an ongoing challenge^29^.

In conclusion, in this study of NAFLD patients enrolled in a randomized controlled trial of a FitBit based structured lifestyle intervention, we accounted for relevant competing and confounding factors to identify independent correlates of HRQOL, dietary habits and motivation to change health behaviors. HRQOL negatively correlated with level of concern for their disease but positively correlated with physical function. Unhealthy dietary patterns were associated with diabetes and younger age. Readiness to change dietary and physical activity habits were closely correlated, indicating that interventions targeted to one behavior change may also impact both health behaviors. We demonstrate potential effectiveness of mobile technology based lifestyle interventions for patients with NAFLD, but highlight challenges in implementation of these programs including fidelity in adherence using step count devices and program retention. Our data suggest future lifestyle interventions in patients with NAFLD may have highest yield if focused on improving physical functioning and tailored dietary counseling based on individual patient comorbidities. Our findings require further validation given attrition rate of participation amidst the COVID pandemic as well as limited duration of the intervention.

## Data Availability

The datasets generated and/or analyzed during the current study are not publicly available due to the small number of patients enrolled in the trial and lack of patient consent to release data but are available from the corresponding author on reasonable request.
